# Vegetarian diet and its possible influence on dental health: A systematic literature review

**DOI:** 10.1111/cdoe.12498

**Published:** 2019-10-01

**Authors:** Kirsten P. J. Smits, Stefan Listl, Milica Jevdjevic

**Affiliations:** ^1^ Department of Dentistry ‐ Quality and Safety of Oral Healthcare Radboud University Medical Center Radboud Institute for Health Sciences Nijmegen The Netherlands; ^2^ Section for Translational Health Economics Department of Conservative Dentistry Heidelberg University Heidelberg Germany

**Keywords:** caries, dental erosion, dental health, systematic literature review, vegetarian diet

## Abstract

**Objectives:**

People following a vegetarian diet could be more prone to oral health problems than people following a nonvegetarian diet. The aim of this systematic review was to examine the possible impacts of following a vegetarian diet on dental hard tissues, focusing on caries development, dental erosion and number of natural teeth.

**Methods:**

PubMed, EMBASE, Web of Science and CINAHL were searched systematically up until 17 April 2019. Original studies comparing dental health (exclusively focusing on dental hard tissues) in vegetarians and nonvegetarians were selected. Study characteristics and outcome data were extracted, and the quality of the studies was assessed using the Newcastle‐Ottawa Scale. When a dental health characteristic was reported in three or more papers in a comparable way, a meta‐analysis was performed.

**Results:**

Twenty‐one papers reporting on 18 studies were included in this review. In meta‐analyses, the vegetarian diet was associated with a higher risk for dental erosion (odds ratio: 2.40 [95% confidence interval: 1.24, 4.66]; *P* = .009) and a lower decayed, missing and filled teeth (DMFT) score (mean difference: −0.15 [95% confidence interval: −0.29, −0.02]; *P* = .023), although the quality of most included studies was poor and the findings for DMFT score became insignificant when only studies on adults were included in the meta‐analysis. A meta‐analysis for the other dental characteristics was not possible due to the limited number of eligible studies. There was inconsistent evidence for a link between following a vegetarian diet and dental caries or the number of natural teeth.

**Conclusions:**

Within the limitations of the present study, the findings suggest that following a vegetarian diet may be associated with a greater risk of dental erosion.

## 1 INTRODUCTION

Vegetarianism is following a diet that is lacking meat, poultry or fish. There are several reasons for following a vegetarian diet, such as health, ethical, environmental or social concerns.^1^ Previously, it was thought that the vegetarian diet would mostly increase the risk of deficiencies, yet over time more and more positive health benefits have also been found. In particular, evidence suggests there is a health‐improving impact of a vegetarian diet on the body mass index, cholesterol levels, glucose levels, risk of cardiovascular disease and cancer.^2^, ^3^, ^4^, ^5^

Dental diseases are highly prevalent worldwide with around 2.5 billion people suffering from untreated caries in their permanent teeth.^6^ These conditions can seriously affect people's well‐being, causing pain and difficulties with eating and speaking. Besides negative impacts on quality of life in both children and adults,^7^, ^8^, ^9^, ^10^ dental diseases impose a considerable economic burden to society with an estimated total worldwide cost of $544 billion in 2015.^11^

To date, it is unclear whether a vegetarian diet may also have impacts on dental health. Several associations have been established for diet and dental health, such as links between sugar consumption and the development of caries^12^, ^13^, ^14^, ^15^ as well as periodontal disease.^16^ Furthermore, there is evidence for a link between consumption of acidic foods and dental erosion.^17^, ^18^, ^19^ However, studies focusing on overall diet patterns and dental diseases are less common. Since the 1970s, some previous studies have examined possible connections between vegetarianism and dental health. The evidence of these studies is mixed; while some studies found positive associations,^20^, ^21^ others found negative associations.^22^, ^23^

To date, and to the best of our knowledge, there has been no systematic overview of the evidence on associations between following a vegetarian diet and dental diseases. Accordingly, the research aim was to systematically review the evidence for associations between a vegetarian diet and (a) noncarious/cervical lesions (NCCL), (b) dental caries and (c) number of natural teeth.

## 2 METHODS

We were interested in observational or intervention studies comparing a vegetarian diet with a nonvegetarian diet in terms of dental health outcomes. A review protocol was written, but not uploaded to a publicly available platform. The review was conducted using the PICOS criteria (Appendix S1).

### 2.1 Exposure of interest

The exposure of interest was the vegetarian diet. There are different variations of the vegetarian diet.^24^ In this study, all diets excluding all meat, poultry or fish were considered as the diet of interest.

### 2.2 Outcomes of interest

The outcomes of interest in this review were diseases of the dental hard tissues. We focused on NCCL, dental caries and number of natural teeth.

Noncarious/cervical lesions was defined as the presence of noncarious or cervical lesions, including dental erosion, dental abrasion and cervical buccal defects. Dental caries was defined as the decayed, missing and filled teeth (DMFT) score or decayed, missing and filled surface (DMFS) score. Whenever the components of DMFT were reported separately, the decayed and filled teeth or surfaces were included in the dental caries group. In addition, outcome measures focusing specifically on the presence of dental caries, (non)visible lesions or white spots of the dental hard tissue were considered. For the number of teeth, we included papers that reported the number of missing or present natural teeth, edentulousness (the complete absence of all natural teeth) and the missing teeth (MT) component of DMFT if reported separately.

### 2.3 Search strategy

The electronic databases PubMed, EMBASE, Web of Science and CINAHL were searched in duplicate and independently for publications up until and including 17 April 2019. The search strategy consisted of headings, subheadings, text words and word variations for oral health, tooth disease, periodontal disease, gingival disease combined with vegetarian or vegetarian diet. The complete search strategies for all databases are shown in Appendix S2. Additionally, the reference list of retrieved studies was screened to identify potential additional publications of relevance.

### 2.4 Study selection and data extraction

Two researchers (KPJS and MJ) independently screened the titles and abstracts of retrieved papers and selected papers for potential inclusion in the review. Full texts were retrieved for the selected papers and read by both researchers to determine eligibility for inclusion.

The data were systematically extracted from the included papers. This included information on the aim, design of study, setting, number of vegetarian and nonvegetarian participants and their characteristics if reported (age, gender and duration of vegetarian diet), dental health outcome, statistical analysis and key study results. Data were extracted by one author (KPJS) and checked by a second author (MJ) using a structured data collection form developed by the researchers. Disagreements were resolved through discussion (KPJS, MJ) and involved a third author (SL) when needed.

### 2.5 Quality assessment of included studies

Papers were checked for risk of bias using the Newcastle‐Ottawa Scale (NOS) for assessing quality of nonrandomized studies^25^ and the adapted NOS for cross‐sectional studies^26^ by two authors independently (KPJS and MJ). The NOS assesses the methodological quality of the study in three domains, the selection of the study groups, the comparability of the groups and the ascertainment of the exposure or outcome of interest. For each domain, stars can be awarded for fulfilling quality requirements. Papers were given a good, fair or poor score for methodological quality based on the NOS scores (Appendix S3).

### 2.6 Statistical analysis

To investigate diet‐related associations with dental health, a meta‐analysis was performed for outcomes that were reported in three or more different studies. Comparable data were extracted and effect sizes with 95% confidence intervals (CI) were calculated. Assuming the presence of heterogeneity across the studies, we used a random effects model. An *I* ^2^ statistic > 50% represented significant heterogeneity. Publication bias was explored via examination of funnel plots. Sensitivity analysis was performed to assess possible associations in children and adults separately. *P*‐values < 0.05 were considered statistically significant. Meta‐analyses were conducted using Stata Special Edition version 15.1 (Stata Corp).

## 3 RESULTS

**Figure 1:**
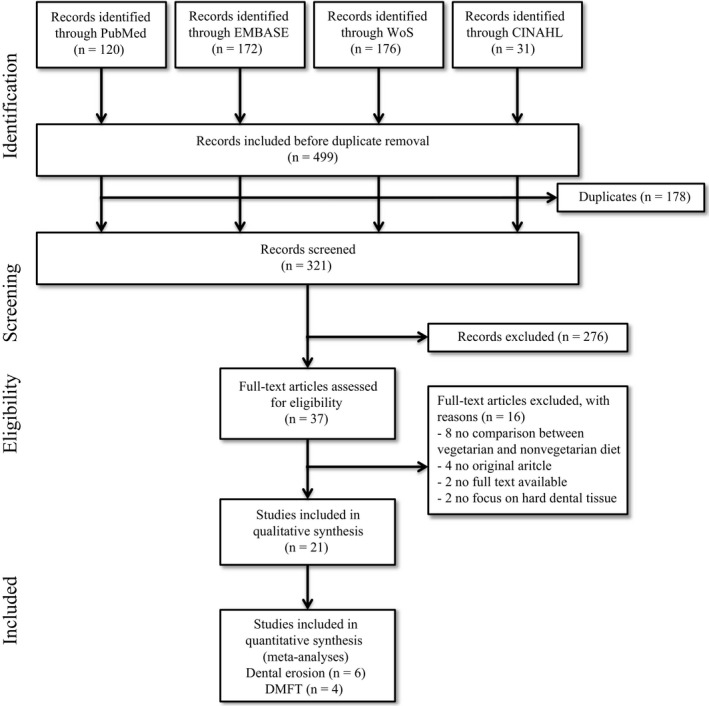
Flow diagram of included studies

The search strategy identified 499 potentially relevant papers, of which 120 papers were retrieved from PubMed, 172 papers from EMBASE, 176 papers from Web of Science and 31 from CINAHL (Figure 1). After removal of duplicates, 321 papers remained for title and abstract screening. Based on the screening, 37 papers were considered for full‐text analysis. Of these 37 papers, 16 did not meet the inclusion criteria. The screening of reference lists of the remaining 21 papers yielded no additional papers fulfilling the inclusion criteria.

### 3.1 Characteristics of included studies

In this review, two papers were reporting similar findings from the same study conducted in Germany,^27^, ^28^ two other papers reporting similar findings from the same Italian study^23^, ^29^ and two reporting on the same study in Trinidad and Tobago.^30^, ^31^ These six papers were considered as three studies. As such, 18 studies were included in this review. Of these, 16 had a cross‐sectional design and two were baseline reports from randomized controlled trials. Most studies were performed in India (n = 9), three studies in Finland, four in other European countries, one in Brazil and one in Trinidad and Tobago. Detailed study characteristics can be found in Appendix S4.

### 3.2 Quality assessment

For assessing the methodological quality of the included studies, the baseline reports of randomized controlled trials were considered to be cross‐sectional studies. According to the NOS, none of the studies was perceived to be of good quality. Two studies reported in three papers^27^, ^28^, ^32^ were perceived to be of fair quality, and the rest was considered being of poor quality. The main reasons were low scoring on sample size, reporting on nonresponders, comparability of the study groups or low scoring on adequate statistical testing.

### 3.3 Dental health outcomes

Quantitative analysis was possible only for the outcomes of dental erosion and for dental caries, specifically the DMFT score. Qualitative analysis was performed for the other dental health outcomes.

#### 3.3.1 NCCL

Eleven different studies reported on NCCL using six different definitions (Appendix S5). The studies reported on the presence of NCCL, presence of dental erosion, presence of tooth wear, severity of tooth wear, presence of dental abrasion and presence of cervical buccal defects.

**Figure 2:**
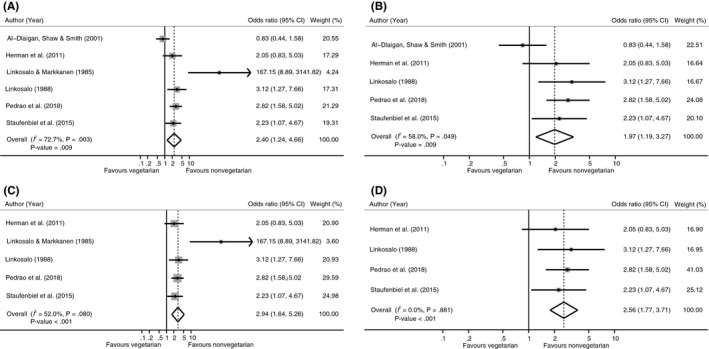
Forest plot of meta‐analysis vegetarian vs nonvegetarian diet with dental erosion. A, Original analysis. B, Sensitivity analysis in adults. C, Sensitivity analysis excluding Linkosalo et al 1985. D, Sensitivity analysis in adults excluding Linkosalo et al 1985

A meta‐analysis was undertaken with the six studies reporting on the presence of dental erosion. The number of included participants per study ranged from 52 to 418. One paper focused on children, while the other five included adults. Despite these differences, the findings showed a significantly higher risk of the presence of dental erosion in vegetarians than in nonvegetarians (odds ratio (OR): 2.40 [95%CI: 1.24, 4.66]; *P* = .009; *I* ^2^ = 72.7%; Figure 2A). Since the effect in Linkosalo & Markkanen (1985)^33^ differed considerably from the effects in other studies and the relative weight was only 4.24%, we performed a sensitivity analysis without this study (Figure 2B). This resulted in a slightly lower but still significant effect (OR: 1.97 [95%CI: 1.19, 3.27]; *P* = .009), the heterogeneity also reduced to *I* ^2^ = 58.0%. Moderate asymmetry was present in both funnel plots (Appendix S6). In addition, we performed a subgroup meta‐analysis including only the studies with adults. The prevalence of tooth erosion was notably higher among adults in the vegetarian group (OR: 2.94 [95%CI: 1.64, 5.26]; *P* < .001; *I* ^2^ = 52.0%; Figure 2C). Exclusion of the Linkosalo & Markkanen (1985)^33^ study led to a pooled OR of 2.56 ([95%CI: 1.77, 3.71]; *P* < .001) with no observed heterogeneity (*I* ^2^ = 0%; Figure 2D).

The other studies showed significantly higher prevalence of NCCL and tooth wear in vegetarians than in nonvegetarians (Appendix S5).

#### 3.3.2 Dental caries

**Figure 3:**
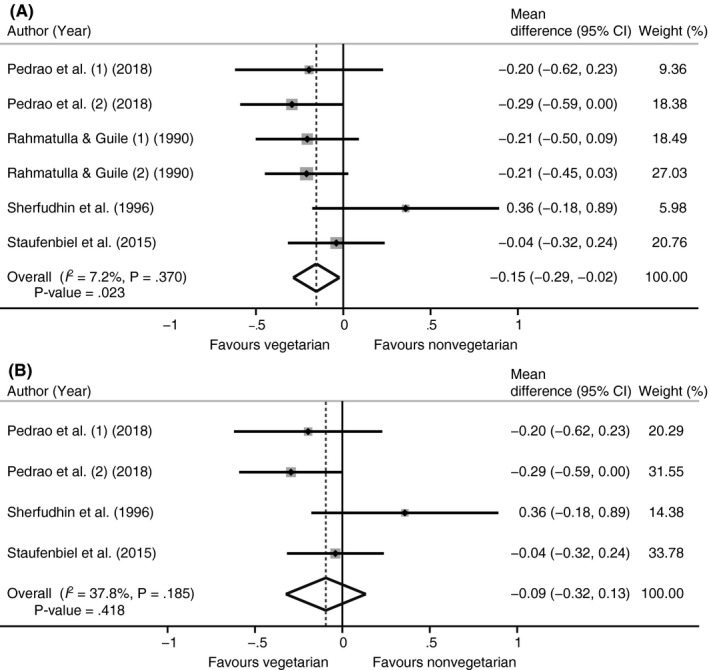
Forest plot of meta‐analysis vegetarian vs nonvegetarian diet with DMFT score. A, Original analysis. B, Sensitivity analysis in adults

Eleven studies reported on dental caries (Appendix S5). In total, 11 different definitions of assessing dental caries were reported. A meta‐analysis using the random effects models was performed on the four studies reporting means and standard deviations on DMFT. For one study, the findings were reported separately for lacto‐ovo‐vegetarians and vegetarians,^32^ while another study reported the findings separately for 12‐ and 15‐year olds.^22^ These groups were included separately in the meta‐analysis as well. The number of included participants per study ranged from 55 to 611. There was a significantly lower mean DMFT score in vegetarians than in nonvegetarians (mean difference: −0.15 [95%CI −0.29, −0.02]; *P* = .023; *I* ^2^ = 7.2%; Figure 3A). A sensitivity analysis including only adults showed a lower and nonsignificant effect (mean difference: −0.10 [95%CI: −0.32, 0.13]; *P* = .418; *I* ^2^ = 37.8%; Figure 3B). Both funnel plots showed no signs of publication bias (Appendix S7). Two of the other three studies reporting on DMFT showed no differences between vegetarian and nonvegetarian children, while the remaining study reported significantly higher mean DMFT scores in vegetarian than in nonvegetarian adults.

Inconsistent findings were reported for the DMFS score. Significantly higher indices were found for decayed surfaces and decayed teeth in vegetarians, as well as higher percentages of decayed and filled surfaces. Two studies suggest lower prevalence of dental caries in vegetarian children than in nonvegetarian children (Appendix S5). Moreover, studies showed that the number of teeth with root caries and the experience of root caries, nonvisible lesions and white spots were significantly higher in vegetarians than in nonvegetarians (Appendix S5).

#### 3.3.3 Number of teeth

Seven studies reported on the number of teeth in relation to the vegetarian diet and showed conflicting findings (Appendix S5). A meta‐analysis was not possible due to inconsistency in outcome definitions. One study found that vegetarians had significantly more teeth than nonvegetarians, while two others were not able to find sizeable differences. Similarly, two studies on missing teeth index as part of the DMFT score found significantly lower index scores in vegetarians, while another did not find differences for vegetarians and nonvegetarians. Finally, one study showed that vegetarians had a higher level of edentulousness than nonvegetarians.

## 4 DISCUSSION

The findings of the meta‐analysis show potential evidence for a twofold greater risk of dental erosion in people following vegetarian diet than in those who were not, although the level of evidence is questionable. High heterogeneity was present; however, performing a subgroup analysis for adults showed an almost threefold greater risk with moderate heterogeneity. In addition, the meta‐analysis on DMFT shows slightly lower scores for vegetarians, with moderate heterogeneity. However, when performed only for an adult subgroup, the effect was not apparent. Furthermore, we found mixed evidence with respect to impacts of vegetarian vs nonvegetarian diets on other measures of dental caries or number of teeth. A quantitative analysis was not possible for these dental outcomes due to limited consistency in the outcome definitions. Due to limited comparability of the studies as well as limited (to no) correction for confounding in the studies, the findings of this review should be interpreted with caution.

Some mechanisms have been proposed that could explain associations between following a vegetarian diet and dental health outcomes. People consuming a vegetarian diet tend to eat more fruits and vegetables than people following a nonvegetarian diet.^34^ Consumption of these acidic foods may lower the pH level in the oral cavity,^35^ which in turn may be related to the development of caries. Shah et al (2004) suggested a possible mechanism whereby people following a vegetarian diet consume too little essential amino acids for maintaining supporting structures healthy or for repair of wear and tear of dental tissues.^36^

However, the association of the vegetarian diet with oral health may be confounded in several ways. The composition of the diet, the lifestyle associated with the vegetarian diet and oral hygiene habits are examples of possible influential factors. The composition of the vegetarian diet may differ depending on time and setting. Some of the studies included in this review have been conducted over 20 years ago, which may have influenced the findings. In addition, the vegetarian diet differs from the nonvegetarian diet in much more food groups than only the meat food group, such as sweets, whole grains and legumes.^21^, ^34^, ^37^ Also, people may follow a vegetarian diet for different reasons (eg for religious beliefs, health concerns, care for environment or any other reason that may be applicable). Therefore, the period of following a vegetarian diet differed between the studies. Possibly following the vegetarian diet from a young age may have a different effect to those following the vegetarian diet only for a few months or years, since dental diseases need some time to manifest. The vegetarian diet is also associated with an healthier lifestyle, that is vegetarians may have a lower BMI, smoke less and may be physically more active than nonvegetarians,^38^ which in turn is related to a better oral health status.^39^, ^40^ Another confounding variable may be the oral hygiene habits, as this may have a major influence on dental health. One study in our review reported significantly better oral hygiene habits among the vegetarian participants, although vegetarians used fluoride‐containing toothpaste less frequently.^28^ In conclusion about the confounding factors, there was little or no reporting on adjustment of confounding variables in the included studies, although some reported adjustment of gender‐, age‐ or diet‐related factors. The lack of adjustment for confounders is a major flaw, and so no hard conclusions can be drawn from the included studies.

### 4.1 Strengths and limitations

The present study is unique and novel because it is, to our knowledge, the first systematic review of the association of vegetarian diets with dental health. An extensive literature search with a standardized, extensive search strategy was performed in four scientific databases. Accordingly, we do believe that we were able to identify all relevant literature available for this systematic review. Our review was not limited to a specific period or geographic area. As such, the included studies were published between 1979 and 2018 and were performed all over the world, although most studies were performed in India. This may have influenced the findings, but we are certain that no important findings were overlooked. A standardized method was used to assess the quality of the methodological aspects in the included studies. Nevertheless, some limitations of the present study should be mentioned. No calibration exercise was done to determine inter‐rater variability. However, agreement was reached for all discrepancies in both the screening and the quality assessment. Overall, the methodological quality of the studies in this systematic literature review (as assessed by the NOS) was poor. Only two out of nineteen studies were considered of fair quality. The NOS is a widely used scale for assessing the methodological quality of studies, although it has some limitations.^41^ Furthermore, only moderate asymmetry was present in the funnel plots and publication bias can therefore not be ruled out. Also, the comparability of the studies was limited due to large differences in dates of publication, settings, study populations and definition of outcome measures. Many different definitions of outcomes were used, making it difficult to compare and group the studies, and to perform meta‐analyses. In addition, the studies largely neglected to adjust for possible confounders that may play a role in the association between the vegetarian diet and dental health.

The main implication of our study comes from the possible twofold greater risk of dental erosion in vegetarians. Given this observation, it seems sensible to recommend raising more awareness of this association among dental professionals and the general public. Our findings are limited and based on studies of mainly lower quality, more and higher quality research (such as longitudinal studies adjusting for possible confounding factors) would be required to confirm the robustness of our findings. If such associations can be confirmed, a next step could be to develop and test interventions for raising awareness among vegetarians and improving their dental health behaviours.

## CONFLICT OF INTEREST

The authors have no conflicting interests to declare. The authors did not receive any financial support for this study. 

## AUTHORS CONTRIBUTION

KPJS and MJ designed research, conducted research, analysed data, performed statistical analysis and wrote the manuscript. SL supervised, mentored and revised the manuscript. KPJS had primary responsibility of final content. All authors read and approved the final manuscript.
