# Supplementary material for: Vegetarian diet and its possible influence on dental health: A systematic literature review
Source: Community Dent Oral Epidemiol. 2019 Oct 1;48(1):7–13. doi: 10.1111/cdoe.12498 (PMC6972589; doi:10.1111/cdoe.12498)
Supplement: Supplementary file 1 [file CDOE-48-7-s001.pdf]

Appendix to 'Vegetarian diet and its possible influence on dental health: A systematic literature review'

Appendix 1. PICOS criteria

**Criteria**	**Definition**
Participants	No restriction on patient population, both children and adults are included. They are allowed to have comorbidities
Interventions	Any vegetarian diet, meaning any diet lacking meat, poultry or fish
Comparisons	Non-vegetarian diet, meaning any diet including meat, poultry or fish
Outcomes	Non-carious or cervical lesions (NCCL), including dental erosion, dental abrasion, and cervical buccal defects<br>Dental caries defined as decayed missing and filled teeth index (DMFT), decayed, missing and filled surface index (DMFS), presence of dental caries, (non-)visible lesions or white spots<br>Number of teeth defined as number of missing or present natural teeth or edentulousness
Study design	Case-control, cross-sectional, cohort, experimental study designs

Appendix 2. Search strategy for both Pubmed and Embase, original to this manuscript

Database	Terms
Pubmed	("Diet, Vegetarian"[Mesh] OR vegetarian* [tiab] OR vegan*[tiab] OR lactovegetarian* [tiab] OR lacto-vegetarian* [tiab] OR lacto-ovovegetarian*[tiab] OR lactoovovegetarian*[tiab] OR Nonmeat[tiab] OR non-meat[tiab] OR “non meat” [tiab] OR “Plant-based”[tiab])<br>AND ("Oral Health"[Mesh] OR "Tooth Diseases"[Mesh] OR "Periodontal Index"[Mesh] OR "Gingival Diseases"[Mesh] OR "Periodontitis"[Mesh] OR oral health [tiab] OR caries[tiab] OR DMF Index[tiab] OR DMFT[tiab] OR DMFS [tiab] OR periodontal disease* [tiab] OR periodontal pocket*[tiab] OR periodontitis [tiab] OR periodontosis[tiab] OR periodontology [tiab] OR tooth[tiab] OR teeth[tiab] OR shortened dental arch [tiab] OR functional dentition [tiab] OR edentul* [tiab] OR plaque index [tiab] OR CPITN [tiab] OR ICDAS [tiab] OR dentin sensitivity[tiab] OR paradontopathy[tiab] OR gingivitis[tiab] OR ((parodontium[tiab] OR peridontium[tiab] OR gingival[tiab]) AND disease*[tiab]) OR ((tooth[tiab] OR teeth[tiab] OR dental[tiab] OR root[tiab]) AND (carious[tiab] OR decay*[tiab] OR lesion*[tiab] OR erosion[tiab] OR disease*[tiab])))
Embase	(exp vegetarian diet/ OR exp vegetarian/ OR vegetarian*.ti,ab,kw. OR vegan*.ti,ab,kw. OR lactovegetarian* .ti,ab,kw. OR lacto-vegetarian* .ti,ab,kw. OR lacto-ovovegetarian*.ti,ab,kw. OR lactoovovegetarian*.ti,ab,kw. OR Nonmeat.ti,ab,kw. OR non-meat.ti,ab,kw. OR non meat.ti,ab,kw. OR Plant-based.ti,ab,kw.)<br>AND (dental health/ OR tooth disease/ or exp dental caries/ or dentin sensitivity/ or exp periodontal disease/ or tooth discoloration/ or exp tooth infection/ or tooth pain/ or tooth pulp disease/ OR oral health .ti,ab,kw. OR caries.ti,ab,kw. OR DMF Index.ti,ab,kw. OR DMFT.ti,ab,kw. OR DMFS .ti,ab,kw. OR periodontal disease* .ti,ab,kw. OR periodontal pocket*.ti,ab,kw. OR periodontitis .ti,ab,kw. OR periodontosis.ti,ab,kw. OR periodontology .ti,ab,kw. OR tooth.ti,ab,kw. OR teeth.ti,ab,kw. OR shortened dental arch .ti,ab,kw. OR functional dentition .ti,ab,kw. OR edentul* .ti,ab,kw. OR plaque index .ti,ab,kw. OR CPITN .ti,ab,kw. OR ICDAS .ti,ab,kw. OR dentin sensitivity.ti,ab,kw. OR paradontopathy.ti,ab,kw. OR gingivitis.ti,ab,kw. OR ((parodontium.ti,ab,kw. OR peridontium.ti,ab,kw. OR gingival.ti,ab,kw.) AND disease*.ti,ab,kw.) OR ((tooth.ti,ab,kw. OR teeth.ti,ab,kw. OR dental.ti,ab,kw. OR root.ti,ab,kw.) AND (carious.ti,ab,kw. OR decay*.ti,ab,kw. OR lesion*.ti,ab,kw. OR erosion.ti,ab,kw. OR disease*.ti,ab,kw.)))

DMF: decayed, missing, filled score; DMFT: decayed, missing, filled teeth score; DMFS: decayed, missing, fillied surfaces score; CPITN: community periodontal index of treatment needs; ICDAS: international caries detection and assessment system.

Appendix 3. Good, fair and poor scores in Newcastle-Ottawa Scale based on number of stars in the three domains: selection of study groups, comparability of study groups and ascertainment of exposure/outcome, original to this manuscript

Score based on NOS for case-control studies	Number of stars				
	Selection of study groups		Comparability of study groups		Ascertainment of exposure/outcome
Good	3 or 4	AND	1 or 2	AND	2 or 3
Fair	2	AND	1 or 2	AND	2 or 3
Poor	0 or 1	OR	0	OR	0 or 1
Score based on NOS for cross-sectional studies					
Good	4 or 5	AND	1 or 2	AND	2 or 3
Fair	2 or 3	AND	1 or 2	AND	2 or 3
Poor	0 or 1	OR	0	OR	0 or 1

NOS: Newcastle-Ottawa scale.

Appendix 4. Extraction forms

Author	Year	Title	Aim	Design
al-Dlaigan Y. H., Shaw L. and Smith A. J.	2001	Vegetarian children and dental erosion	To assess the prevalence of vegetarian children, to determine the prevalence of dental erosion in these children and to see if there were any differences between vegetarian and non-vegetarian children in the prevalence of dental erosion	Cross-sectional
Chandra S. and Chawla T. N.	1979	Incidence of dental caries in Lucknow school-going children	To assess the incidence of dental caries in school going children (?)	Cross-sectional
Chopra A., Rao N. C., Gupta N., Vashisth S. and Lakhanpal M.	2015	The Predisposing Factors between Dental Caries and Deviations from Normal Weight	To investigate specifically the correlation between BMI, diet, and dental caries among 12-15-year-old school-going children	Cross-sectional
Herman K., Czajczynska-Waszkiewicz A., Kowalczyk-Zajac M. and Dobrzynski M.	2011	Assessment of the influence of vegetarian diet on the occurrence of erosive and abrasive cavities in hard tooth tissues	To investigate the influence of vegetarian diet on the occurence of abrasive and erosive cavities	Cross-sectional
Khan A. A., Jain S. K. and Shrivastav A.	2008	Prevalence of Dental Caries among the Population of Gwalior (India) in Relation of Different Associated Factors	To assess the pattern of caries in relation to type of caries, age group, gender and dietary habit	Cross-sectional
Kumar S., Kumar A., Debnath N., Kumar A., Badiyani B.K., Basak D., Ali M.S.A. and Ismail M.B.	2015	Prevalence and risk factors for non-carious cervical lesions in children attending special needs schools in India	To examine the prevalence and risk factors of noncarious cervical lesions	Cross-sectional

Appendix 4. Extraction forms

Author	Year	Title	Aim	Design
Kumar M., Verma R., Bansal M., Singh S., Rehan S., Kumar V., Simran Dr.	2018	To evaluate the severity, distribution of occlusal tooth wear and its correlation with bite force in young North Indian adults	To detemine the severity and distribution of occlusal tooth wear among young North Indian adults and to evaluate the correlation of occlusal tooth wear with bith force	Cross-sectional
Laffranchi L., Zotti F., Bonetti S., Dalessandri D. and Fontana P.	2010	Oral implications of the vegan diet: observational study	To investigate the oral implications of a vegan diet	Baseline analysis RCT
Linkosalo E. and Markkanen H.	1985	Dental erosions in relation to lactovegetarian diet	To investigate the frequency and severity of erosions associated with diet of lactovegetarians and to analyze which factors in the lactovegetarian diet and saliva are related to dental erosions	Cross-sectional
Linkosalo E., Ohtonen S., Markkanen H., Karinpaa A. and Kumpusalo E.	1985	Caries, periodontal status and some salivary factors in lactovegetarians	To sompare the dental and periodontal health and some salivary factors of lactovegetarians and their sex- and age-matched controls	Cross-sectional
Linkosalo E.	1988	Dietary habits and dental health in Finnish Seventh-Day Adventists	To compare the eating habits of Finnish Seventh-Day Adventists with people having a traditional omnivorous diet	Cross-sectional

Appendix 4. Extraction forms

Author	Year	Title	Aim	Design
Padrão A.M.N., Andrews Portes L., Padilha Gomes E., Figueira Teixeira F.C., da Costa Pereira A., de Oliveira N.C.	2018	Erosive Tooth Wear and Dietary Petters: A Clinical Study	To assess the influence of dietary pattern (vegetarian, lacto-ovo vegetarian and omnivore) on erosive tooth wear	Cross-sectional
Rafeek R. N., Marchan S., Eder A. and Smith W. A.	2006	Tooth surface loss in adult subjects attending a university dental clinic in Trinidad	To determine the prevalence of tooth surface loss and to investigate the relationship to tooth brushing, medical history, parafunction and dietary habits	Cross-sectional
Rahmatulla M. and Guile E. E.	1990	Relationship between dental caries and vegetarian and non-vegetarian diets	To explore the relationship between the level of caries among vegetarians and non-vegetarians	Cross-sectional
Shah N., Parkash H. and Sunderam K. R.	2004	Edentulousness, denture wear and denture needs of Indian elderly--a community-based study	To evaluate the level of edentulousness, denture wear habits and denture needs of the elderly	Cross-sectional
Sherfudhin H., Abdullah A., Shaik H. and Johansson A.	1996	Some aspects of dental health in young adult Indian vegetarians. A pilot study	To investigate dietary habits, caries prevalence, and dentoalveolar characteristics, including tooth wear, in vegetarian and non-vegetarian young adults	Cross-sectional

Appendix 4. Extraction forms

Author	Year	Title	Aim	Design
Smith W. A., Marchan S. and Rafeek R. N.	2008	The prevalence and severity of non-carious cervical lesions in a group of patients attending a university hospital in Trinidad	To determine the prevalence oand severity of all forms of NCCLs and to investigate the relationship between medical and dental histories, oral hygiene practices, dietary habits and occlusion	Cross-sectional
Staufenbiel I., Weinspach K., Forster G., Geurtsen W. and Gunay H.	2013	Periodontal conditions in vegetarians: a clinical study	To investigate the influence of a vegetarian diet on periodontal parameters	Baseline analysis RCT
Staufenbiel I., Adam K., Deac A., Geurtsen W. and Gunay H.	2015	Influence of fruit consumption and fluoride application on the prevalence of caries and erosion in vegetarians--a controlled clinical trial	To compare the prevalence of dental caries and erosian in vegetarian and non-vegetarian subjects	Baseline analysis RCT
Venugopal T., Kulkarni V. S., Nerurker R. A., Damle S. G. and Patnekar P. N.	1998	Epidemiological study of dental caries	To relate dietary habits like type of diet, sweet consumption, feeding habits and level of nutrition to prevalence of dental caries	Cross-sectional
Zotti F., Laffranchi L., Fontana P., Dalessandri D. and Bonetti S.	2014	Effects of fluorotherapy on oral changes caused by a vegan diet	To measure changes in the presence and size of white spots after 1 year of daily sodium fluoride application. Changes in pH, DMFt, plaque index and gingival index were evaluated	Baseline analysis RCT

Appendix 4. Extraction forms

Author	Year	Country	Setting	Population	Age	Age category	Vegetarians
al-Dlaigan Y. H., Shaw L. and Smith A. J.	2001	UK	12 schools in South-Birmingham	418 children	14 years	Children	42 Self-reported
Chandra S. and Chawla T. N.	1979	India	Schools in Lucknow	15000 children	4-15 years	Children	8776 Self-reported
Chopra A., Rao N. C., Gupta N., Vashisth S. and Lakhanpal M.	2015	India	Schools in four blocks of Panchkula	810 children	12-15 years	Children	Unclear
Herman K., Czajczynska-Waszkiewicz A., Kowalczyk-Zajac M. and Dobrzynski M.	2011	Poland	Subjects were recruited throuigh advertisement on the internet and in magazines for vegetarians, most of them inhabited Wroclaw and neighbouring villages	92 adults	17-51 years	Adults	46
Khan A. A., Jain S. K. and Shrivastav A.	2008	India	Dentistry department of Gajra Raja Medical College, Gwalior and various other local district hospitals of Gwalior	104 patients	5-72 years	All	97 (unclear)
Kumar S., Kumar A., Debnath N., Kumar A., Badiyani B.K., Basak D., Ali M.S.A. and Ismail M.B.	2015	India	Special needs schools in Indore city	393 children	12-15 years	Children	167 Self-reported

Appendix 4. Extraction forms

Author	Year	Country	Setting	Population	Age	Age category	Vegetarians
Kumar M., Verma R., Bansal M., Singh S., Rehan S., Kumar V., Simran Dr.	2018	Inda	Patient attending the Out Patient Department of different private clinics of North India.	164 adults	20-40 years	Adults	66 self-reported
Laffranchi L., Zotti F., Bonetti S., Dalessandri D. and Fontana P.	2010	Italy	Northern Italy	30 adults	24-60 years	Adults	15 (vegan)
Linkosalo E. and Markkanen H.	1985	Finland	?	52 adults	39.6 ± 10.2	Adults	26 Self-reported
Linkosalo E., Ohtonen S., Markkanen H., Karinpaa A. and Kumpusalo E.	1985	Finland	?	56 adults	? (age groups: ≤35; 36-44; ≥45)	Adults	28 Self-reported
Linkosalo E.	1988	Finland	Annual summer convention of the Finnish Seventh-Day Adventists	237 adults	18-86 years	Adults	123 Seventh-Day Adventists

Appendix 4. Extraction forms

Author	Year	Country	Setting	Population	Age	Age category	Vegetarians
Padrão A.M.N., Andrews Portes L., Padilha Gomes E., Figueira Teixeira F.C., da Costa Pereira A., de Oliveira N.C.	2018	Brazil	Seventh-day Adventists Churches in metropolitan region of São Paulo	207 adults	35-74 years	Adults and elderly	125 vegetarian/lacto-ovo vegetarisn
Rafeek R. N., Marchan S., Eder A. and Smith W. A.	2006	Trinidad and Tobago	University of the West Indies, School of Dentistry Polyclinic	155 adults	16 to 73	Adults and elderly	11 Self-reported
Rahmatulla M. and Guile E. E.	1990	India	Random sample of schools in Tamil Nadu, South India	1200 children (12yr (n=589) and 15yr (n=611))	12 and 15 years	Children	48 12-year-olds and 77 15-year-olds
Shah N., Parkash H. and Sunderam K. R.	2004	India	Urban and rural areas with Geriatric Health Care Programme run by a non-govermental organization in Vasant Kunj in South Delhi	1240 elderly	≥60 years	Elderly	769 Self-reported
Sherfudhin H., Abdullah A., Shaik H. and Johansson A.	1996	India	Students at Saveetha Dental College, Madras	55 adults	17-27 years	Adults	30 Hindu vegetarians

Appendix 4. Extraction forms

Author	Year	Country	Setting	Population	Age	Age category	Vegetarians
Smith W. A., Marchan S. and Rafeek R. N.	2008	Trinidad and Tobago	University of the West Indies, School of Dentistry adult dental clinic	156 patients	16 to 73 years, mean 40.6	Adults and elderly	?
Staufenbiel I., Weinspach K., Forster G., Geurtsen W. and Gunay H.	2013	Germany	?	200 patients	20-81 years	Adults and elderly	100 (11 vegans)
Staufenbiel I., Adam K., Deac A., Geurtsen W. and Gunay H.	2015	Germany	?	200 patients	20-81 years	Adults and elderly	100 (11 vegans)
Venugopal T., Kulkarni V. S., Nerurker R. A., Damle S. G. and Patnekar P. N.	1998	India	Pediatric out-patient department, school clinic and well-baby clinic in Bombay	2000 children	<16 years	Children	906 Self-reported
Zotti F., Laffranchi L., Fontana P., Dalessandri D. and Bonetti S.	2014	Italy	Northern Italy	100 adults	24-60 years	Adults	50 vegans

Appendix 4. Extraction forms

Author	Year	Nonvegetarians	Duration of vegetarian diet	Oral health outcome
al-Dlaigan Y. H., Shaw L. and Smith A. J.	2001	376 Self-reported		Presence of dental erosion
Chandra S. and Chawla T. N.	1979	6224 Self-reported		DMFT, DT, MT, FT
Chopra A., Rao N. C., Gupta N., Vashisth S. and Lakhanpal M.	2015	Unclear		DMFT
Herman K., Czajczynska-Waszkiewicz A., Kowalczyk-Zajac M. and Dobrzynski M.	2011	46 matched on sex, age and education	1-18 years (9 on average)	Presence of dental erosion, presence of dental abrasion
Khan A. A., Jain S. K. and Shrivastav A.	2008	7 (unclear)		Presence of dental caries
Kumar S., Kumar A., Debnath N., Kumar A., Badiyani B.K., Basak D., Ali M.S.A. and Ismail M.B.	2015	216 Self-reported		Presence of non-carious cervical lesions

Appendix 4. Extraction forms

Author	Year	Nonvegetarians	Duration of vegetarian diet	Oral health outcome
Kumar M., Verma R., Bansal M., Singh S., Rehan S., Kumar V., Simran Dr.	2018	98 self-reported		Severity of tooth wear
Laffranchi L., Zotti F., Bonetti S., Dalessandri D. and Fontana P.	2010	15	18 months - 20 years	DMFT, presence of non-visible lesions, presence of white spots
Linkosalo E. and Markkanen H.	1985	26 controls	Minimum 2 years, 6.1±5.3	Presence of dental erosion
Linkosalo E., Ohtonen S., Markkanen H., Karinpaa A. and Kumpusalo E.	1985	28 matched on age and gender	At least 2 years	Number of natural teeth, precentage decayed or filled surfaces of surfaces at risk
Linkosalo E.	1988	114 matched on age and sex		Presence of dental erosion, number of natural teeth, percentage of decayed or filled surfaces of surfaces at risk, DMFS

Appendix 4. Extraction forms

Author	Year	Nonvegetarians	Duration of vegetarian diet	Oral health outcome
Padrão A.M.N., Andrews Portes L., Padilha Gomes E., Figueira Teixeira F.C., da Costa Pereira A., de Oliveira N.C.	2018	82 omnivores		DMFT, MT, presence of dental erosion
Rafeek R. N., Marchan S., Eder A. and Smith W. A.	2006	144 Self-reported		Presence of tooth wear
Rahmatulla M. and Guile E. E.	1990	541 12-year-olds and 534 15-year-olds		Presence of caries, DMFT
Shah N., Parkash H. and Sunderam K. R.	2004	471 Self-reported		Number of natural teeth
Sherfudhin H., Abdullah A., Shaik H. and Johansson A.	1996	25 matched on age and gender	From birth or early childhood until present	Number of natural teeth, DMFT, presence of tooth wear, presence ofcervical buccal defects

Appendix 4. Extraction forms

Author	Year	Nonvegetarians	Duration of vegetarian diet	Oral health outcome
Smith W. A., Marchan S. and Rafeek R. N.	2008	?		Presence of non-carious cervical lesions
Staufenbiel I., Weinspach K., Forster G., Geurtsen W. and Gunay H.	2013	100 matched on age, gender and smoking habits		DMFT, DMFS, DT, MT, FT, presence of dental erosion
Staufenbiel I., Adam K., Deac A., Geurtsen W. and Gunay H.	2015	100 matched on age, gender and smoking habits		DMFT, DMFS, DT, FT, DS, FS, presence of dental erosion, presence of root caries
Venugopal T., Kulkarni V. S., Nerurker R. A., Damle S. G. and Patnekar P. N.	1998	1094 Self-reported		Presence of dental caries
Zotti F., Laffranchi L., Fontana P., Dalessandri D. and Bonetti S.	2014	50 non-vegans		DMFT, presence of non-visible lesions, presence of white spots

Appendix 4. Extraction forms

**Author**	**Year**	**Statistical analysis/confounding variables**	**Result**
al-Dlaigan Y. H., Shaw L. and Smith A. J.	2001	t-test for the prevalence of dental erosion and Chi-square analysis for the severity of dental erosion; no adjustment for confounding variables	No significant differences for the prevalence or severity of erosion between vegetarians and non-vegetarian children
Chandra S. and Chawla T. N.	1979	Not mentioned/not tested	The incidence of caries was more in vegetarian than in non-vegetarian, although no statistical method was used to confirm significant differences between the two groups.
Chopra A., Rao N. C., Gupta N., Vashisth S. and Lakhanpal M.	2015	Not mentioned/not tested	The vegetarians had a non significant higher DMFT (1.72) as compared to mixed diet children (1.70)
Herman K., Czajczynska-Waszkiewicz A., Kowalczyk-Zajac M. and Dobrzynski M.	2011	Mann-Whitney U-test and Chi-square. Logistic regression (odds ratios) with adjustment for length of diet and frequency of consumption of particular products	No significant differences for erosive occurence between vegetarians and non-vegetarians, odds ratios did not show significant results.
Khan A. A., Jain S. K. and Shrivastav A.	2008	Not mentioned/not tested	Highest prevalence of caries in patients with vegetarian diet
Kumar S., Kumar A., Debnath N., Kumar A., Badiyani B.K., Basak D., Ali M.S.A. and Ismail M.B.	2015	Chi-square analysis, frequency distribution analysis, logistic regression; no adjustment for confounding variables	Significant higher risk (OR 1.86) to develop non-carious cervical lesions when consuming vegetarian diet compared to mixed diet

Appendix 4. Extraction forms

Author	Year	Statistical analysis/confounding variables	Result
Kumar M., Verma R., Bansal M., Singh S., Rehan S., Kumar V., Simran Dr.	2018	Mann-Whitney U test; no adjustment for confounding variables	No statistical significant differences in the tooth wear score between vegetarians (32.76±13.75) en non-vegetarians (35.12±13.95)
Laffranchi L., Zotti F., Bonetti S., Dalessandri D. and Fontana P.	2010	Non-parametric Wilcoxon rank-sum test for DMFT, Fisher's exact test for presence of non-visible lesions and white spots; no adjustment for confounding variables	Presence of non-visible lesions and white spots was significantly higher in vegan population compared to omnivores. DMFT did not differ significantly.
Linkosalo E. and Markkanen H.	1985	Student's t-test and linear multiple regression analysis. Included variables (analysis is performed only in lactovegetarians) are saliva flow rate, consumption of vinegar, consumption of citrus fruits, saliva phosphosrus, saliva buffering capacity, age and coarse fresh food.	Dental erosion was present in lactovegetarians, but not in omnivore subjects
Linkosalo E., Ohtonen S., Markkanen H., Karinpaa A. and Kumpusalo E.	1985	Student's t-test; no adjustment for confounding variables	No statistically significant differences were found in either the number of remaining teeth and caries or in periodontal status
Linkosalo E.	1988	Student's t-test and Chi-square; no adjustment for confounding variables	The mean value of DMFS was lower in the SDA group compared to controls ($p<0.001$). The SDA group had a higher number of natural teeth ($p<0.001$) as well as a higher percentage of DF surfaces of surfaces at risk. The SDA group had a higher prevalence of erosion ($p<0.01$)

Appendix 4. Extraction forms

Author	Year	Statistical analysis/confounding variables	Result
Padrão A.M.N., Andrews Portes L., Padilha Gomes E., Figueira Teixeira F.C., da Costa Pereira A., de Oliveira N.C.	2018	ANOVA with Tukey's post-hoc test, Kruskal-Wallis with Dunn's post-hoc test, Fisher's exact test, odds ratios, multiple logistic regression analysis; adjustment for gender	No statistical differences in DMFT scores, MT was significantly higher in omnivore group than in lacto-ovo vegetarian group (p=0.027). Vegetarian and lacto-ovo vegetarian group showed more dental erosive tooth wear than the omnivore group (p<0.05)
Rafeek R. N., Marchan S., Eder A. and Smith W. A.	2006	Odds ratios; no adjustment for confounding variables	No significant higher risk of tooth surface loss (OR: 279 (95% CI 0.76-10.27)) when consuming vegetarian diet compared to mixed diet
Rahmatulla M. and Guile E. E.	1990	Fourway tables to calculate Chi-square with Yates' correction and Odds ratios; no adjustment for confounding	There were significant associations between vegetarian diet and less dental caries
Shah N., Parkash H. and Sunderam K. R.	2004	Chi-square analysis; no adjustment for confounding variables	Higher level of edentulousness in those consuming vegetarian diet compared to non-vegetarian diet (not significantly correlated)
Sherfudhin H., Abdullah A., Shaik H. and Johansson A.	1996	Mann-Whitney U-test and for DMFT partial correlation with gender as controlling variable	Number of natural teeth and DMFT were not significantly different between vegetarians and non-vegetarians. The dentition wear index was significantly higher in vegetarians (p<0.01). 30% of vegetarians showed cervical defects, while 12% of non-vegetarians shows cervical defects

Appendix 4. Extraction forms

Author	Year	Statistical analysis/confounding variables	Result
Smith W. A., Marchan S. and Rafeek R. N.	2008	Odds ratios; no adjustment for confounding variables	A significant association between risk of NCCLs and patients who were vegetarian (although OR: 6.7 (95% CI 0.8-53.8)
Staufenbiel I., Weinspach K., Forster G., Geurtsen W. and Gunay H.	2013	Mann-Whitney test, no confouding variables included; multivariate regression analysis with gender, age, vegetarian, HI, DMFT, smoking habits, frequency of oral hygiene procedures, frequency of dentist visit and level of edication included (not significant for vegetarian diet)	Vegetarians showed more eroded and decayed teeth than non-vegetarians.
Staufenbiel I., Adam K., Deac A., Geurtsen W. and Gunay H.	2015	t-test for DMFT and DMFS. Mann-Whitney test for DT, FT, DS, FS and number of teeth with dental erosion. No adjustment for confounding variables	DMFT and DMFS were not significantly different between vegetarians and nonvegetarians. But, vegetarians showed significantly more decayed teeth and surfaces and a higher number of teeth with dental erosion.
Venugopal T., Kulkarni V. S., Nerurker R. A., Damle S. G. and Patnekar P. N.	1998	Not mentioned/not tested	Caries prevalence was lower in children with vegetarien diet than with non-vegetarian diet
Zotti F., Laffranchi L., Fontana P., Dalessandri D. and Bonetti S.	2014	Non-parametric Wilcoxon rank-sum test for DMFT, Fisher's exact test for presence of non-visible lesions and white spots; no adjustment for confounding variables	More non-visible lesions and more white spots in vegans compared to omnivores. DMFT did not show significant differences

Appendix 4. Extraction forms

Author	Year	NOS for cohort studies<br>Representativeness of sample	Sample size	Non-respondents	Ascertainment of exposure	Comparability
al-Dlaigan Y. H., Shaw L. and Smith A. J.	2001	a	a	b	b	0
Chandra S. and Chawla T. N.	1979	a	b	c	b	0
Chopra A., Rao N. C., Gupta N., Vashisth S. and Lakhanpal M.	2015	a	a	c	c	0
Herman K., Czajczynska-Waszkiewicz A., Kowalczyk-Zajac M. and Dobrzynski M.	2011	a	b	c	c	2
Khan A. A., Jain S. K. and Shrivastav A.	2008	a	b	c	c	0
Kumar S., Kumar A., Debnath N., Kumar A., Badiyani B.K., Basak D., Ali M.S.A. and Ismail M.B.	2015	a	a	c	c	0

Appendix 4. Extraction forms

Author	Year	NOS for cohort studies<br>Representativeness of sample	Sample size	Non-respondents	Ascertainment of exposure	Comparability
Kumar M., Verma R., Bansal M., Singh S., Rehan S., Kumar V., Simran Dr.	2018	a	a	c	b	0
Laffranchi L., Zotti F., Bonetti S., Dalessandri D. and Fontana P.	2010	d	b	c	b	2
Linkosalo E. and Markkanen H.	1985	c	b	c	b	2
Linkosalo E., Ohtonen S., Markkanen H., Karinpaa A. and Kumpusalo E.	1985	c	b	c	b	2
Linkosalo E.	1988	a	b	c	c	2

Appendix 4. Extraction forms

Author	Year	NOS for cohort studies<br>Representativeness of sample	Sample size	Non-respondents	Ascertainment of exposure	Comparability
Padrão A.M.N., Andrews Portes L., Padilha Gomes E., Figueira Teixeira F.C., da Costa Pereira A., de Oliveira N.C.	2018	b	b	b	a	2
Rafeek R. N., Marchan S., Eder A. and Smith W. A.	2006	b	b	c	b	0
Rahmatulla M. and Guile E. E.	1990	a	b	c	c	1
Shah N., Parkash H. and Sunderam K. R.	2004	b	a	c	b	0
Sherfudhin H., Abdullah A., Shaik H. and Johansson A.	1996	c	b	c	b	2

Appendix 4. Extraction forms

Author	Year	**NOS for cohort studies**<br>**Representativeness of sample**	**Sample size**	**Non-respondents**	**Ascertainment of exposure**	**Comparability**
Smith W. A., Marchan S. and Rafeek R. N.	2008	a	a	c	c	0
Staufenbiel I., Weinspach K., Forster G., Geurtsen W. and Gunay H.	2013	d	a	c	b	2
Staufenbiel I., Adam K., Deac A., Geurtsen W. and Gunay H.	2015	d	a	c	b	2
Venugopal T., Kulkarni V. S., Nerurker R. A., Damle S. G. and Patnekar P. N.	1998	b	c	c	b	0
Zotti F., Laffranchi L., Fontana P., Dalessandri D. and Bonetti S.	2014	d	b	c	b	2
		a=1, b=1, c=0, d=0	a=1, b=0	a=1, b=0, c=0	a=2, b=1, c=0	control most important factor = 1, additional factor = 2

Appendix 4. Extraction forms

Author	Year	NOS for cohort studies<br>Assessment of outcome	Statistical test	Rating (poor, fair, good)
al-Dlaigan Y. H., Shaw L. and Smith A. J.	2001	a	b	poor
Chandra S. and Chawla T. N.	1979	a	b	poor
Chopra A., Rao N. C., Gupta N., Vashisth S. and Lakhanpal M.	2015	a	b	poor
Herman K., Czajczynska-Waszkiewicz A., Kowalczyk-Zajac M. and Dobrzynski M.	2011	a	a	poor
Khan A. A., Jain S. K. and Shrivastav A.	2008	a	b	poor
Kumar S., Kumar A., Debnath N., Kumar A., Badiyani B.K., Basak D., Ali M.S.A. and Ismail M.B.	2015	a	a	poor

Appendix 4. Extraction forms

Author	Year	NOS for cohort studies<br>Assessment of outcome	Statistical test	Rating (poor, fair, good)
Kumar M., Verma R., Bansal M., Singh S., Rehan S., Kumar V., Simran Dr.	2018	a	b	poor
Laffranchi L., Zotti F., Bonetti S., Dalessandri D. and Fontana P.	2010	a	b	poor
Linkosalo E. and Markkanen H.	1985	d	b	poor
Linkosalo E., Ohtonen S., Markkanen H., Karinpaa A. and Kumpusalo E.	1985	d	b	poor
Linkosalo E.	1988	d	b	poor

Appendix 4. Extraction forms

Author	Year	NOS for cohort studies<br>Assessment of outcome	Statistical test	Rating (poor, fair, good)
Padrão A.M.N., Andrews Portes L., Padilha Gomes E., Figueira Teixeira F.C., da Costa Pereira A., de Oliveira N.C.	2018	a	a	fair
Rafeek R. N., Marchan S., Eder A. and Smith W. A.	2006	a	b	poor
Rahmatulla M. and Guile E. E.	1990	a	a	poor
Shah N., Parkash H. and Sunderam K. R.	2004	a	a	poor
Sherfudhin H., Abdullah A., Shaik H. and Johansson A.	1996	a	b	poor

Appendix 4. Extraction forms

Author	Year	NOS for cohort studies<br>Assessment of outcome	Statistical test	Rating (poor, fair, good)
Smith W. A., Marchan S. and Rafeek R. N.	2008	a	a	poor
Staufenbiel I., Weinspach K., Forster G., Geurtsen W. and Gunay H.	2013	a	a	fair
Staufenbiel I., Adam K., Deac A., Geurtsen W. and Gunay H.	2015	a	a	fair
Venugopal T., Kulkarni V. S., Nerurker R. A., Damle S. G. and Patnekar P. N.	1998	a	b	poor
Zotti F., Laffranchi L., Fontana P., Dalessandri D. and Bonetti S.	2014	a	b	poor
		a=2, b=2, c=1, d=0	a=1, b=0	

Appendix 5. Funnel plot meta-analysis of vegetarian diet with dental erosion

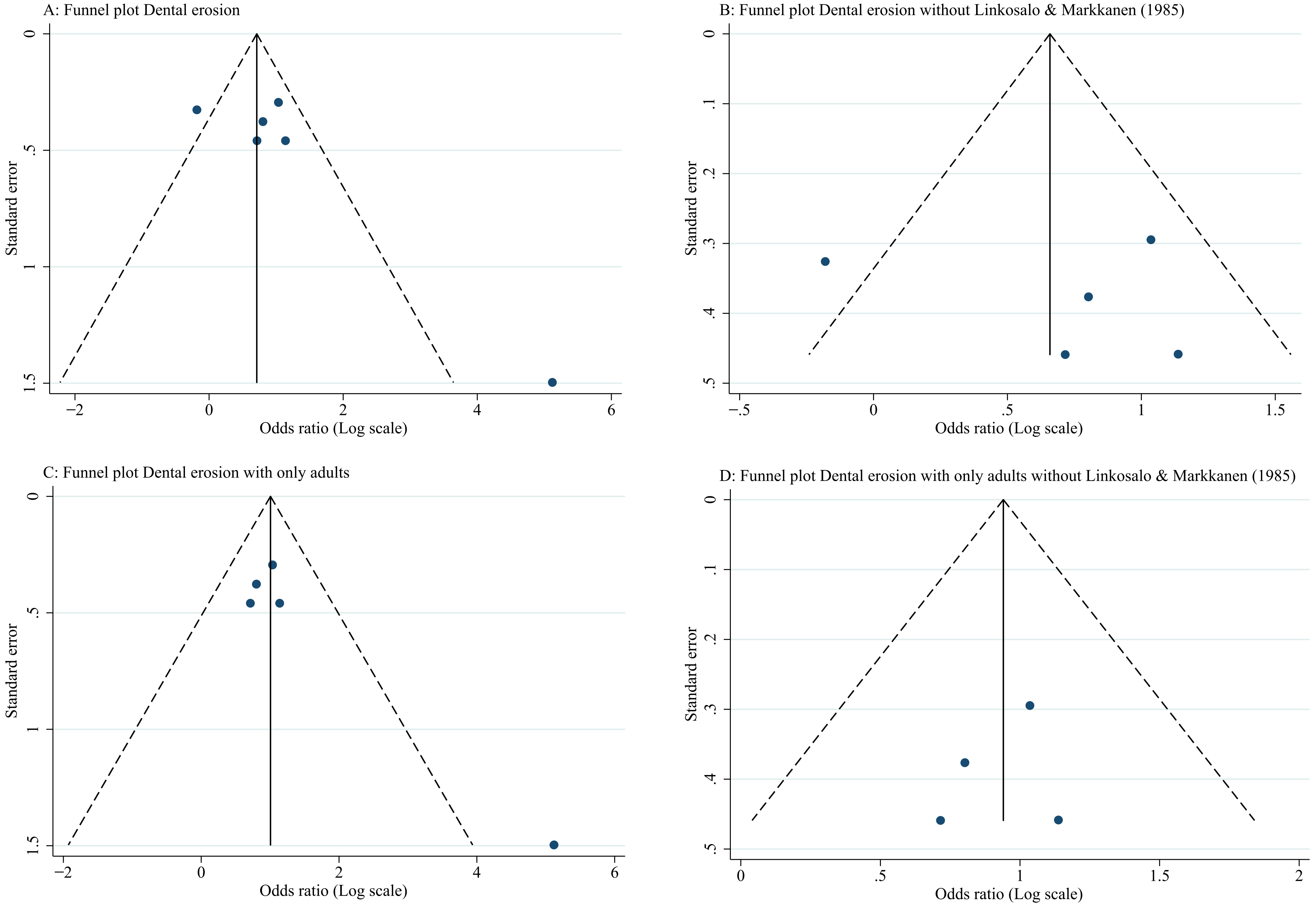

Appendix 6. Funnel plot meta-analysis of vegetarian diet with DMFT

A: Funnel plot DMFT

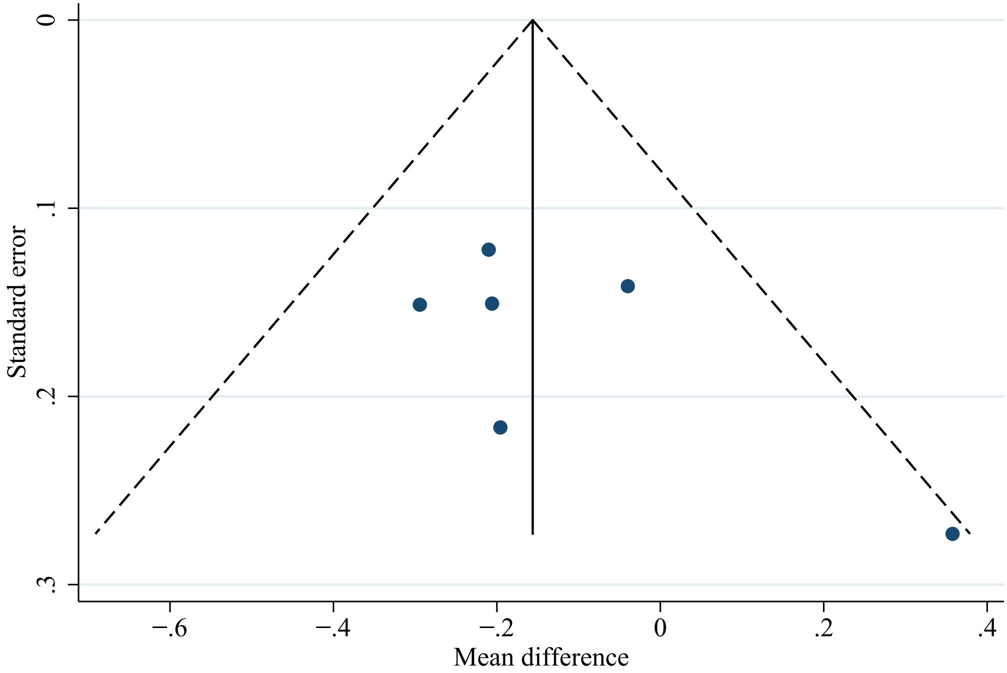

B: Funnel plot DMFT with only adults

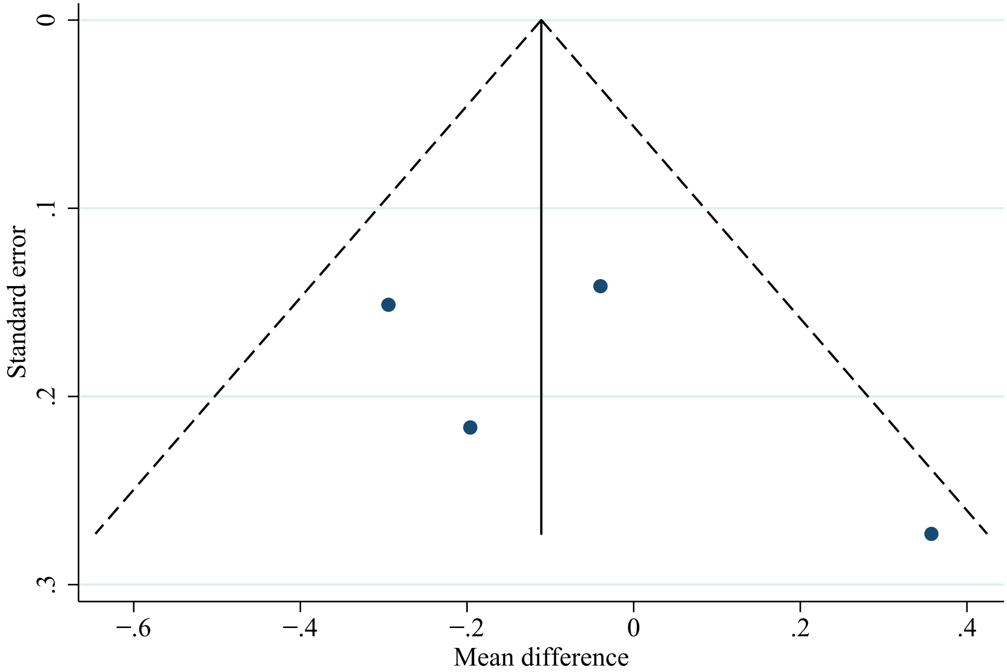

Appendix 7. Reference list of included studies

al-Dlaigan YH, Shaw L, Smith AJ. Vegetarian children and dental erosion. Int J Paediatr Dent. 2001;11(3):184-192.

Chandra S, Chawla TN. Incidence of dental caries in Lucknow school-going children. J Indian Dent Assoc. 1979;51(4):109-110.

Chopra A, Rao NC, Gupta N, Vashisth S, Lakhanpal M. The Predisposing Factors between Dental Caries and Deviations from Normal Weight. N Am J Med Sci. 2015;7(4):151-159.

Herman K, Czajczynska-Waszkiewicz A, Kowalczyk-Zajac M, Dobrzynski M. Assessment of the influence of vegetarian diet on the occurrence of erosive and abrasive cavities in hard tooth tissues. Postepy Hig Med Dosw (Online). 2011;65:764-769.

Khan AA, Jain SK, Shrivastav A. Prevalence of Dental Caries among the Population of Gwalior (India) in Relation of Different Associated Factors. Eur J Dent. 2008;2(2):81-85.

Kumar S, Kumar A, Debnath N, et al. Prevalence and risk factors for non-carious cervical lesions in children attending special needs schools in India. J Oral Sci. 2015;57(1):37-43.

Kumar M, Verma R, Bansal M, et al. To Evaluate the Severity, Distribution of Occlusal Tooth Wear and its Correlation with Bite Force in Young North Indian Adults. Open Dent J. 2018;12:735-741.

Laffranchi L, Zotti F, Bonetti S, Dalessandri D, Fontana P. Oral implications of the vegan diet: observational study. Minerva Stomatol. 2010;59(11-12):583-591.

Linkosalo E, Ohtonen S, Markkanen H, Karinpaa A, Kumpusalo E. Caries, periodontal status and some salivary factors in lactovegetarians. Scand J Dent Res. 1985;93(4):304-308.

Linkosalo E, Markkanen H. Dental erosions in relation to lactovegetarian diet. Scand J Dent Res. 1985;93(5):436-441.

Linkosalo E. Dietary habits and dental health in Finnish Seventh-Day Adventists. Proc Finn Dent Soc. 1988;84(2):109-115.

Pedrão AMN, Andrews Portes L, Padilha Gomes E, Figueira Teixeira FC, da Costa Pereira A, de Oliveira NC. Erosive Tooth Wear and Dietary Patterns: A Clinical Study. Oral Health Prev Dent. 2018;16(2):145-151.

Rafeek RN, Marchan S, Eder A, Smith WA. Tooth surface loss in adult subjects attending a university dental clinic in Trinidad. Int Dent J. 2006;56(4):181-186.

Rahmatulla M, Guile EE. Relationship between dental caries and vegetarian and non-vegetarian diets. Community Dent Oral Epidemiol. 1990;18(5):277-278.

Shah N, Parkash H, Sunderam KR. Edentulousness, denture wear and denture needs of Indian elderly--a community-based study. J Oral Rehabil. 2004;31(5):467-476.

Sherfudhin H, Abdullah A, Shaik H, Johansson A. Some aspects of dental health in young adult Indian vegetarians. A pilot study. Acta Odontol Scand. 1996;54(1):44-48.

Smith WA, Marchan S, Rafeek RN. The prevalence and severity of non-carious cervical lesions in a group of patients attending a university hospital in Trinidad. J Oral Rehabil. 2008;35(2):128-134.

Staufenbiel I, Weinspach K, Forster G, Geurtsen W, Gunay H. Periodontal conditions in vegetarians: a clinical study. Eur J Clin Nutr. 2013;67(8):836-840.

Staufenbiel I, Adam K, Deac A, Geurtsen W, Gunay H. Influence of fruit consumption and fluoride application on the prevalence of caries and erosion in vegetarians--a controlled clinical trial. Eur J Clin Nutr. 2015;69(10):1156-1160.

Venugopal T, Kulkarni VS, Nerurker RA, Damle SG, Patnekar PN. Epidemiological study of dental caries. Indian J Pediatr. 1998;65(6):883-889.

Zotti F, Laffranchi L, Fontana P, Dalessandri D, Bonetti S. Effects of fluorotherapy on oral changes caused by a vegan diet. Minerva Stomatol. 2014;63(5):179-188.
